# Removal of 2,4-Dichlorophenolyxacetic acid (2,4-D) herbicide in the aqueous phase using modified granular activated carbon

**DOI:** 10.1186/2052-336X-12-28

**Published:** 2014-01-10

**Authors:** Mansooreh Dehghani, Simin Nasseri, Mojtaba Karamimanesh

**Affiliations:** 1 Department of Environmental Health Engineering, School of Health, Shiraz University of Medical Sciences, Shiraz, Iran; 2 Department of Environmental Health Engineering, School of Public Health, and Center for Water Quality Research, Institute for Environmental Research, Tehran University of Medical Sciences, Tehran, Iran

**Keywords:** Herbicide, 2,4-D, Adsorption, Aqueous phase, Kinetics

## Abstract

**Background:**

Low cost 2,4-Dichlorophenolyxacetic acid (2,4-D) widely used in controlling broad-leafed weeds is frequently detected in water resources. The main objectives of this research were focused on evaluating the feasibility of using granular activated carbon modified with acid to remove 2,4-D from aqueous phase, determining its removal efficiency and assessing the adsorption kinetics.

**Results:**

The present study was conducted at bench-scale method. The influence of different pH (3–9), the effect of contact time (3–90 min), the amount of adsorbent (0.1-0.4 g), and herbicide initial concentration (0.5-3 ppm) on 2,4-D removal efficiency by the granular activated carbon were investigated. Based on the data obtained in the present study, pH of 3 and contact time of 60 min is optimal for 2,4-D removal. 2,4-D reduction rate increased rapidly by the addition of the adsorbent and decreased by herbicide initial concentration (63%). The percent of 2,4-D reduction were significantly enhanced by decreasing pH and increasing the contact time. The adsorption of 2,4-D onto the granular activated carbon conformed to Langmuir and Freundlich models, but was best fitted to type II Langmuir model (R^2^ = 0.999). The second order kinetics was the best for the adsorption of 2,4-D by modified granular activated carbon with R^2^ > 0.99. Regression analysis showed that all of the variables in the process have been statistically significant effect (p < 0.001).

**Conclusions:**

In conclusion, granular activated carbon modified with acid is an appropriate method for reducing the herbicide in the polluted water resources.

## Background

2,4-Dichlorophenolyxacetic acid (2,4-D) known as a phenoxy compound generally has a low acute toxicity with low to moderate mobility in soil [1-5]. It is widely used for post-emergence for selective control of broad-leafed weeds in wheat, maize, rice and many other crops in Fars province of Iran [6,7]. 2,4-D is found in mixed formulations. The consumption rate of 2,4-D is 50–100 liters per hectare. 2,4-D is a synthetic auxin, absorbed well through the leaves.

In 1987, the International Agency for Research on Cancer (IARC) concluded that 2,4-D is a B-2 carcinogen [8]. Recently, 2,4-D is classified as a group D carcinogen by USEPA and a suspected endocrine disrupter [9]. Other than cancer, increase in abnormal sperms, sperms immobility and death, increase of lymphocytes, probability of immune deficiency disorders, and incidence of nervous, kidney, and respiratory diseases are among the concerns associated with using this herbicide. Fetus mortality, urinary system disorders, and congenital diseases have also been observed in the exposed animals [10].

The dissipation of 2,4-D in soil was found to follow the first-order kinetics. 2,4-D herbicide is relatively non-persistent and degrades rapidly in soil. The herbicide had a short half-life in soil (between 1.5 and 16 days). 2,4-D half-life is greatly influenced by soil type, temperature, soil pH and moisture. Despite its short half-life in soil compare to other herbicide [11], it has been detected in water resources [12]. Due to very high water solubility of 2,4-D (900 mg/L), low soil adsorption coefficient, it can be detected in surface and groundwater and also in the finished drinking water of many countries [13]. It is regulated by USEPA with a maximum contaminant level (MCL) of 0.07 mg/L for drinking water [9].

In general, herbicide can be removed through various methods, such as photocatalytic degradation [14], ultrasound technology [15,16], electrocoagulation process [17], combined photo-Fenton and biological oxidation [18-21] and nanofiltration [22]. Nowadays, adsorption process is widely used for the treatment of the waters contaminated by insecticides [23], dyes [24-26] and phenols [27]. The most advantages of adsorption technique include effectiveness even at low contaminant concentrations, selectivity, regenerability, and cost efficiency [28]. Moreover, activated carbon, either powdered or granular, is considered as an effective adsorbent for removing the organic contaminant in the aqueous environments due to its porous structure and large specific surface area, high removal efficiency and the feasibility of using in large scales [3,7]. However, one of the limitations of using the adsorption process is the transfer of pollutions from one media to another. Therefore, another method should be developed to remove the pollutions from the adsorbent. Numerous studies have demonstrated that the presence of natural and synthetic organic matter in the water resources can significantly reduce the adsorption capacity of granular activated carbon. Therefore, background water quality affects its removal efficiency. Moreover, desorption is an important phenomena when designing adsorption process to remove the pollutions [29].

The removal efficiency by adsorption process is mostly affected by various factors, including pH, contact time and the initial concentration of the contaminant [6]. The study by Salman and Hameed [6] indicated that granular activated carbon (F300) was a reliable adsorbent for 2,4-D and carbofuran removal and fitted well by pseudo-second-order adsorption kinetics. According to Langmuir and Ferundlich isotherm models, the maximum adsorption capacity of 2,4-D and carbofuran was 182.82 and 96.15 mg per g adsorbent, respectively [6]. Aksu and Kabasakal [7] showed that 2,4-D adsorption kinetics in aqueous environments using granular activated carbon closely followed Ferundlich and Kolbe-Corrigan models. In addition, the maximum adsorption rate; i.e., 518 mg per g activated carbon, was obtained at pH = 2 at 45°C [7]. Moreno et al. also conducted a study and showed that the removal of S-triazine herbicides (propazine, prometrin, and prometon) from water was accomplished using three types of inexpensive granular activated carbon and its adsorption kinetics followed Temkin isotherm [30].

Since Fars is an agricultural province of Iran and enjoys the top rank in wheat and corn production in the country in recent years, herbicides especially 2,4-D has been widely used as a selective herbicide to control broad-leaf and grassy weeds in agricultural wheat and corn fields. Moreover, there is a concern regarding the contamination of water resources and its effect on people’s health and the environment. Therefore, the objectives of the study were to (i) evaluate the feasibility of using granular activated carbon in removing 2,4-D in the aqueous phase, (ii) determine the optimum conditions so that the standard limit can be achieved, and (iii) assess the kinetics and mechanisms of herbicide adsorption on activated carbon.

## Methods

The adsorption experiments were carried out in triplicates at the bench-scale method. The study parameters were pH, contact time, adsorbent dose, and initial herbicide concentration. Factorial design was used for the analysis of the parameters and their interaction effects were studied as well. To reduce the scatter in the data, log of transformation and geometric mean were used.

### The preparation of the modified activated carbon

Granular activated carbon with effective size of 1.5 mm (No. 1.0214.1000) was used in this study. In order to increase the adsorbent ratio, granular activated carbon was washed with double distilled water for several times. Then, it was soaked in 0.1 normal HCL under laboratory conditions for 24 hours. Afterwards, it was rinsed with double distilled water for several times and dried in the oven at 105°C for 24 hours. The modified activated carbon was kept in the desiccators.

### Chemicals and analytical method

All chemicals were purchased from Merck (Germany). 2,4-D standard (98% purity) was supplied by Sigma-Aldrich Company USA, St. Louis Missouri. For 2,4-D detection a Agilent 1200 Model high performance liquid chromatography (USA) system with a C_18_ column (5 μm particles, 250 mm length, and 4.6 mm internal diameter) was calibrated and tested prior to injection of the samples. The mobile phase consisting of water-acetonitrile (8:92 ratio) was used. A UVDAD detector at the wavelength of 244 nm was used to detect 2,4-D in the samples after extraction through an SPE (Solid Phase Extraction) cartridge [31,32]. The retention time for the 2,4-D herbicide was 2.150 min. The detection limit for 2,4-D was 1 ng/L.

### Extraction of 2,4-D from the aqueous phase

In order to separate the solid phase, 3 ml MeOH was passed through SPE cartridge containing 500 mg C_18_. Then, 3 ml deionized water was passed through the column. Finally, 3 ml deionized water with pH = 3 adjusted by phosphoric acid was passed through the column. Afterwards, the column was washed twice with two 500 μl MeOH aliquot and 2,4-D herbicide was extracted from the column. The herbicide was then dried by nitrogen gas. After that, the herbicide was dissolved in 500 μl acetonitrile and it was analyzed using HPLC.

### Effects of pH and contact time on the rate of adsorption of 2,4-D by modified granular activated carbon

To measure the influence of pH on the adsorption of 2,4-D by granular activated carbon in the aqueous phase, different pH from 3–9 (interval of 2) with three replications was used at the herbicide concentration of 2 ppm, adsorbent dose of 0.2 g per 50 ml deionized water, and the contact time of 90 min on a reciprocal shaker (150 rpm) at room temperature (20°C). A blank without activated carbon was also used for all the experiments. After that, the residual of 2,4-D was measured.

To measure the influence of contact time on the adsorption of 2,4-D by granular activated carbon in the aqueous phase, various contact times (3–90 min) were evaluated with three replications at the optimum pH, the herbicide concentration of 2 ppm, adsorbent dose of 0.2 g per 50 ml deionized water on a reciprocal shaker (150 rpm) at room temperature (20°C).

### Effects of adsorbent dose and initial herbicide concentration on the rate of adsorption of 2,4-D by modified granular activated carbon

In order to determine the effect of adsorbent dose on the adsorption of 2,4-D by granular activated carbon in the aqueous phase, various activated carbon doses (0–2.1 g) were evaluated with three replications at the optimum pH and contact time, the herbicide concentration of 2 ppm on a reciprocal shaker (150 rpm) at room temperature (20°C).

Different herbicide concentrations (0.5-3 mg/L) were used to determine their effect on the adsorption rate at the optimum pH, contact time and adsorbent dose on a reciprocal shaker (150 rpm) at room temperature (20°C). All the experiments were performed in three replications.

### Adsorption isotherm

Four adsorption isotherms (Freundlich, Langmuir type I, Langmuir type II and Temkin) are examined for 2,4-D adsorption data on the modified granular activated carbon. According to the 2,4-D adsorption data, the fit of Freundlich, Langmuir and Temkin isotherms have been determined by drawing the logarithmic equilibrium concentration in the liquid and solid phase.

Ferundlich isotherm is empirical equation and the adsorption energy is related to heterogeneity of sorption sites of the adsorbent. Adsorption isotherm parameters were calculated for 2,4-D using the Freundlich equation [33,34]:

(1) logqe=logkf+1nlogCe

Where:

q_e_ (mg/kg) is the amount of 2,4-D adsorbed per kg of soil. C_e_ (mg/L) is the equilibrium concentration in solution; K_f_ (L/kg) and 1/n are the adsorption coefficients and the adsorption constant, respectively.

Langmuir isotherm is the most widely applied adsorption isotherm which is used for adsorption of dissolved substances in the aqueous phase. The isotherm is based on monolayer adsorption on the adsorbent with homogeneous adsorption energy [35]. Temkin isotherm includes a factor that takes into account the adsorbent-adsorbate interaction [35,36]. The Langmuir equation (2) and the Temkin equation (3) were also used to fit the adsorption data:

(2) $$\frac{C_{e}}{q_{e}}=\frac{1}{Kq_{m}}+\frac{C_{e}}{q_{m}}$$

(3) $$q_{e}=B_{1}1nk_{t}+B_{1}1nC_{e}$$

Where:

q_m_ is the required amount of herbicide for forming one layer (mg/g). K, K_t_ and B_1_ are adsorption constants, and q_e_ and C_e_ are as defined above.

### Adsorption kinetics

The kinetics of 2,4-D adsorption on modified granular activate carbon was studied by two common models, namely, first order and second order models.

The first order and second order kinetic models are expressed by the following equations (4, 5):

(4) $$\log\left(q_{e}-q_{t}\right)=\log\left(q_{e}\right)-\frac{k_{1}}{2.303}t$$

(5) $$\frac{t}{q_{t}}=\left(\frac{1}{k_{2}q_{e}^{2}}\right)+\left(\frac{1}{q_{e}}\right)t$$

Where:

K_1_ (mol.g^-1^.min^-1^) is the rate constant for the first order sorption, K_2_ (g.mol^-1^.min^-1^) is the rate constant of second order sorption and q_e_ (mol.g^-1^) is the amount of herbicide adsorbed at time t.

## Results

### Effects of pH and contact time on the rate of adsorption of 2,4-D by modified granular activated carbon

**Figure 1:**
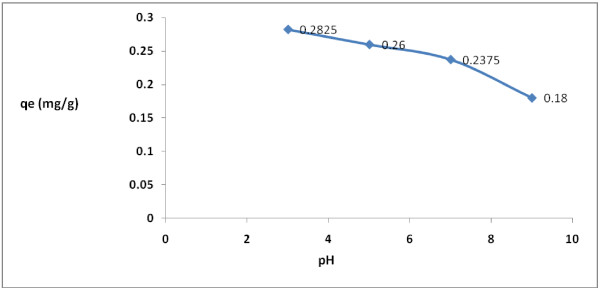
Effect of pH on 2,4-D removal by modified granular activated carbon.

The variations of pH on the rate of adsorption of 2,4-D by modified granular activated carbon were shown on Figure 1. According to Figure 1, the maximum adsorption capacity of 2,4-D by modified granular activated carbon (0.2825 mg/g) and the reduction of 56.5% was achieved in an acidic range (pH = 3). In basic pH levels (pH = 9), on the other hand, the adsorption capacity of the herbicide in the aqueous phase decreased (0.18 mg/g). According to regression analysis it can be concluded that there was a significant difference between pH and 2,4-D adsorption rate (p < 0.001).

**Figure 2:**
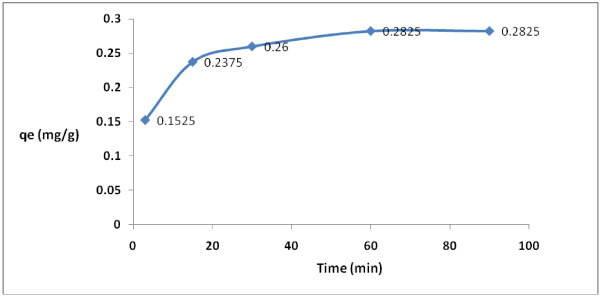
Effect of time on 2,4-D removal by modified granular activated carbon.

As shown in Figure 2, the rate of adsorption of 2,4-D by modified granular activated carbon increased as the contact time increased (3–60 min). After 60 min equilibration time, its rate became constant (60–90 min). Regression analysis showed that there was a significant difference between contact time and 2,4-D adsorption rate (p < 0.001).

### Effects of adsorbent dose and initial herbicide concentration on the rate of adsorption of 2,4-D by modified granular activated carbon

**Figure 3:**
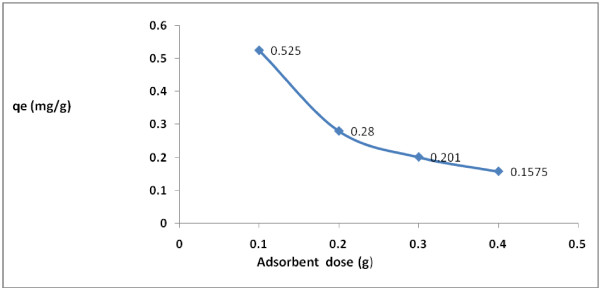
Effect of adsorbent dose on 2,4-D removal by modified granular activated carbon.

2,4-D adsorption rate increased as the applied adsorbent dose increased. According to Figure 3, 2,4-D reduction rate and the adsorption capacity for different adsorbent dose was in the range of 52.5 to 63% and 0.525 to 0.1575 mg/g, respectively. According to regression analysis it can be concluded that there was a significant difference between catalyst dose and 2,4-D adsorption removal rate (p < 0.001).

The effect of initial herbicide concentration on 2,4-D adsorption rate was shown in Table 1. The adsorption capacity was in the range of 0.085 to 0.385 mg/g. According to data obtained in the current study, 2,4-D adsorption rate decreased from 68 to 51.33% as the initial herbicide concentration increased from 0.5 to 3 ppm. Regression analysis showed that there was a significant difference between initial herbicide concentration and the 2,4-D adsorption removal rate (p < 0.001).

**Table 1:** The effect of initial 2,4-D concentration on its rate of adsorption by modified granular activated carbon

**pH = 3 contact time = 60 min adsorbent dose = 4 g/L**									
**Initial concentration (mg/L)**	**Residual 2,4-D (mg/L)**	**Efficiency (%)**	**q**_**e **_**(mg/g)**	**1/Ce**	**1/qe**	**C**_**e**_**/q**_**e**_	**LnC**_**e**_	**Log C**_**e**_	**Log q**_**e**_
0.5	0.16	68	0.085	6.25	11.76471	1.882353	-1.83258	-0.79588	-1.07058
1	0.36	64	0.16	2.777778	6.25	2.25	-1.02165	-0.4437	-0.79588
2	0.85	57.5	0.2875	1.176471	3.478261	2.956522	-0.16252	-0.07058	-0.54136
3	1.46	51.33	0.385	0.684932	2.597403	3.792208	0.378436	0.164353	-0.41454

**Figure 4:**
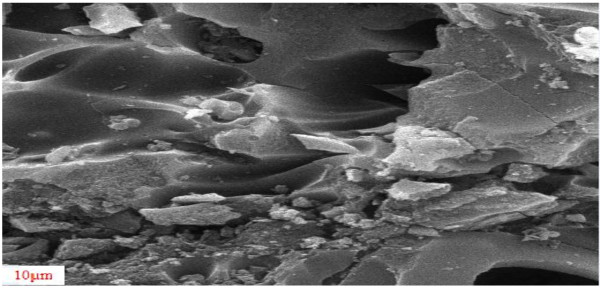
SEM image of modified granular activated carbon.

**Figure 5:**
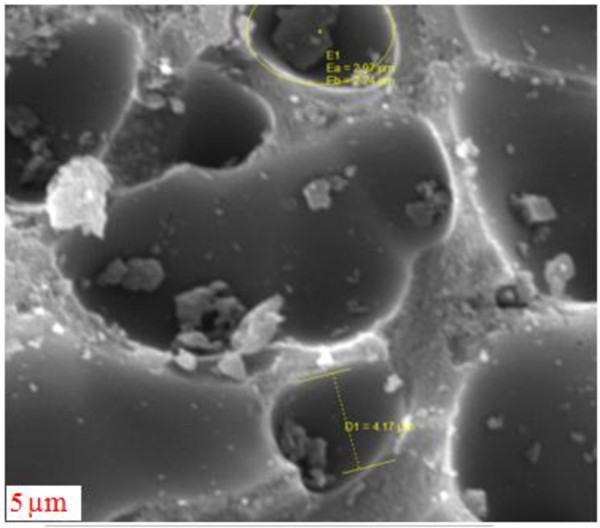
SEM image of modified granular activated carbon.

Scanning electron microscope (SEM) images of granular activated carbon modified by acid are presented in Figures 4 and 5.

### Adsorption isotherms

**Figure 6:**
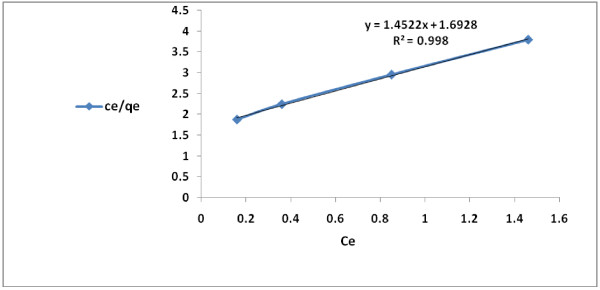
Langmuir (Type I) isotherm for 2,4-D adsorption by modified granular activated carbon.

**Figure 7:**
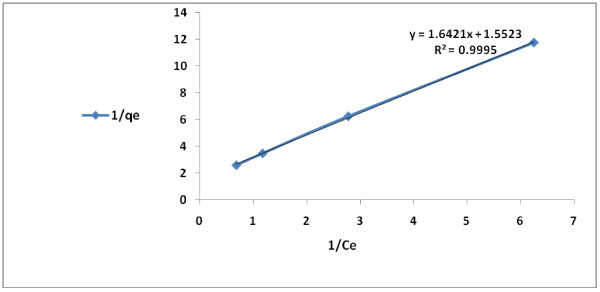
Type II Langmuir (Type II) isotherm for 2,4-D adsorption by modified granular activated carbon.

**Figure 8:**
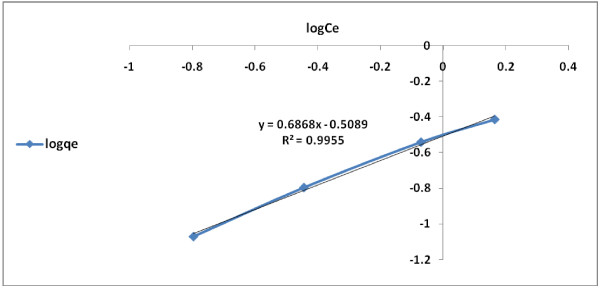
Ferundlich isotherm for 2,4-D adsorption by modified granular activated carbon.

**Figure 9:**
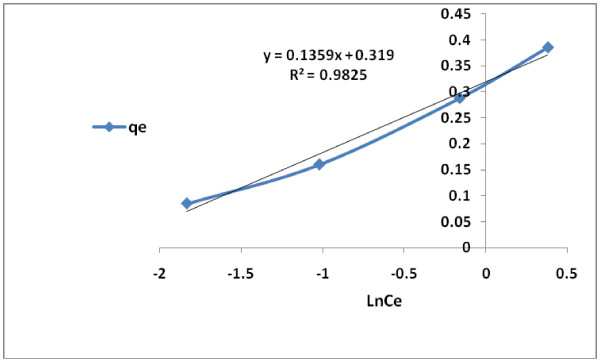
Temkin isotherm for 2,4-D adsorption by modified granular activated carbon.

The conformity of adsorption data for the modified granular activated carbon to Freundlich, Langmuir and Temkin isotherms (Table 2) are indicated by the coefficient of determination (R^2^). The 2,4-D adsorption data described well to Langmuir and Freundlich isotherms, respectively. However, the fit to Langmuir (type II) adsorption model (R^2^ = 0.999) was greater than that of Freundlich (R^2^ = 0.995) or Temkin (R^2^ = 0.982) (Figures 6, 7, 8 and 9). On the basis of the measured R^2^ value, the 2,4-D adsorption conformity to different isotherms can be arranged in the following order:

**Table 2:** Parameters of 2,4-D adsorption isotherm on modified granular activated carbon

**Langmuir (І) isotherm**			**Freundlich isotherm**		
q_m_ (mg/g)	K_L_ (L/mg)	R^2^	K_f_	n	R^2^
0.688	0.858	0.998	3.22	1.457	0.995
**Langmuir (ІІ) isotherm**			**Temkin isotherm**		
q_m_ (mg/g)	K_L_ (L/mg)	R^2^	K_t_	B_t_	R^2^
0.644	0.945	0.999	10.622	0.135	0.982

Langmuir (type II) adsorption > Langmuir (type I) > adsorption Freundlich adsorption > Temkin adsorption

## Discussion

Adsorption technique is widely used for the removal of toxic organic compounds. pH is one of the most important parameters affecting the adsorption process and has played a major role in the adsorption studies [37]. pH also controls the process of adsorption on the adsorbent due to the electrostatic force of attraction during the adsorption process [38].

Data regarding the adsorption of 2,4-D herbicide showed that more than 60 percent of 2,4-D reduction was obtained at pH = 3.0 by granular activated carbon due to the improvement of the adsorbent’s characteristics in the acidic pH [39]. However, the rate of adsorption of 2,4-D decreased at higher pH levels due to the formation of oxygen-containing species on the surface of granular activated carbon. In fact, formation of these species on the adsorbent surface leads to lower accessibility of the adsorption sites to 2,4-D molecules and, consequently, reduced its adsorption capacity [40]. Similar results were also obtained by TaghiZadeh et al. [41]. 2,4-D is a weak acid with pK_a_ = 2.73 [42,43]. At higher pH values, the herbicide is converted rapidly to the anion (negatively charged) form which has a negative impact on the adsorption rate. It can be concluded that at higher pH values the electrostatic repulsion force or diffusion of the herbicide ions at the surface of granular activated carbon was increased, therefore the equilibrium adsorption decreased as pH rise above 3.0. In general, any pH changes in a solution may change the molecular characteristics of the pesticides and affect its adsorption. Aksu and Kabasakal (2004) also reported the same results regarding the effect of pH on the rate of adsorption of 2,4-D using granular activated carbon in the aqueous phase [7]. Similar results were also obtained on the adsorption of 2,4-D and carbofuran using compost and industrial wastes. They reported pH range (2.2-6.6) was optimal pH to succeed 70-80% removal efficiency. However, a significant decrease was observed in the adsorption rate at pH more than 12 [2].

Determining the equilibration time is another important factor to achieve the maximum rate of herbicide adsorption in the aqueous phase (2). According to the results illustrated in the current study, at first the adsorption rate of 2,4-D increases very fast as the contact time increases. After that, its rate becomes slow and then constant (Figure 2). This phenomenon may be related to the presence of many vacant adsorption sites on the adsorbent surface. After that, the remaining sites are not easily accessible for the target molecules. Therefore, it can be concluded that the high reduction of 2,4-D by granular activated carbon at the beginning of the adsorption process might be due to the presence of many available vacant adsorption sites for the adsorption. Then, the adsorption rate is basically controlled by the migration rate of the contaminant [44]. This phenomenon was also reported by many other studies [41,45,46]. Dehghani et al. [47] also found that the adsorption kinetics of atrazine in soil had an initial steep slope reaching a plateau with a relative slow equilibration. Presumably initial quick adsorption is a surface phenomenon, followed by a slow migration and diffusion of the compound into the organic matter and solid texture [47]. Therefore, adsorption studies are very important and make it possible to evaluate retention of the herbicide by adsorbent. In addition, understanding sorption kinetics allows prediction of how fast such a reaction reaches equilibrium and the possible mechanisms involved. Kinetic studies may provide important information about the herbicide fate in the aqueous phase.

The herbicide initial concentration plays a major role in overcoming mass transfer resistance of the adsorbate between aqueous and solid phase [38]. Present study was performed at a very low concentration of 2,4-D herbicide, the concentration normally was detect in water resources, whereas other study using higher initial concentration of the herbicide found the opposite effect [48]. Data obtained in this research demonstrated that as the initial herbicide concentration increased, the rate of adsorption reduced. Higher available binding sites may result in higher rate of herbicide adsorption. Our results agree with Malakoutian et al. and Aksu and Kabasakal findings [7,49].

According to the findings of the current study, the adsorbent dose is an important parameter for determining the rate of 2,4-D adsorption. Figure 3 depicts, 2,4-D removal was dependent on the adsorbent dose in the solution and the herbicide removal increased with the increase in the adsorbent dose (Figure 3). The result showed that as the adsorbent concentration increased, the percentage of herbicide removal increased as well. The same results obtained by Aksu and Kabasakal [7].

The adsorption capacity of modified granular activated carbon is strongly dependent of initial 2,4-D concentration. Therefore, applying the adsorption process is more likely to be cost-effective in the situation where the initial 2,4-D concentration is relatively low. The relative cost for the adsorption process estimated to be lower compare to other technology such as advanced oxidation process and membrane technology. In summary, the benefits of using modified granular activated carbon for 2,4-D treatment are simple technology, easy to implement and commercially available with low capital and installation costs.

Over a limited range of 2,4-D concentration, Langmuir adsorption isotherm is observed for the herbicide, which is highly soluble and shows a relatively high K_ow_ value. Many other researchers have found that 2,4-D adsorption conformed to Langmuir isotherm [6,7]. The conformity of the data to various isotherms can be compared using correlation coefficient (R^2^) [50]. The adsorption coefficient (q_m_) and the adsorption constant (K_L_) for Langmuir adsorption isotherm for 2,4-D are 0.644 (mg/g) and 0.945 L/mg, respectively. At the equilibrium, the fit to Langmuir isotherm (R^2^ = 0.999) was more than Ferundlich isotherm (R^2^ = 0.995). Langmuir isotherm model is utilized for monolayer adsorption with a limited number of adsorption sites.

Kinetic models were also examined with obtained data. The coefficients of correlation as well as the kinetic parameters of 2,4-D adsorption on modified granular activated carbon are presented in Table 3. From the data obtained in this research, the correlation coefficient of the first order kinetic for the adsorption of 2,4-D was (R^2^ =0.966). However, the correlation coefficient of the second order kinetic for the adsorption was greater than 0.99. Therefore, the adsorption process of herbicide on modified granular activated carbon follows the second order rate kinetic.

**Table 3:** First order and second order kinetics parameters for the adsorption of 2,4-D on modified granular activated carbon

**First order kinetic**			**Second order kinetic**		
q_e_	K_1_	R^2^	K_2_	q_e_	R^2^
7.030	0.0621	0.966	1.338	0.282	0.999

## Conclusion

In conclusion, the results reported in the adsorption of 2,4-D by the modified granular activated carbon revealed that 2,4-D in liquid phase was effectively and rapidly retained by the solid phase. The rate of 2,4-D adsorption showed an initial increase, reaching a plateau with a relative slow rate. The adsorption of 2,4-D was increased with decreasing initial concentration of 2,4-D and increasing the adsorbent dose. Therefore, 2,4-D adsorption in the aqueous solution was relatively high at pH = 3 and contact time = 60 min. Although, 2,4-D adsorption data showed a highly significant fit to Langmuir, Freundlich, and Temkin isotherms, the fit to Langmuir isotherm proved to be more suitable, as compared with Freundlich and/or Temkin. The reduction rate of 2,4-D from aqueous solutions was more than 68% at optimal conditions. According to data obtained in the current study, the level of 2,4-D concentration using modified granular activated carbon was not exceeded the standard limit for drinking water. Therefore, the modified granular activated carbon can be used as efficient and cost-effective method to remove 2,4-D from water resources. It is highly recommended that the dynamic column testing using modified granular activated carbon to remove 2,4-D for different background water qualities to be performed.

## Competing interests

The authors declare that they have no competing interests.

## Authors’ contributions

The overall implementation of this study including design, experiments and data analysis, and manuscript preparation were the results of the corresponding author’s efforts. All authors have made extensive contribution into the review and finalization of this manuscript. All authors read and approved the final manuscript.
